# A pathway away from measurement‐based patient‐specific QA: A review of contemporary quality assurance for external‐beam radiotherapy sub‐systems

**DOI:** 10.1002/acm2.70407

**Published:** 2025-12-02

**Authors:** Michael Barnes

**Affiliations:** ^1^ Department of Radiation Oncology Calvary Mater Hospital Newcastle Newcastle New South Wales Australia

**Keywords:** data transfer QA, linac QA, patient specific QA, PSQA, quality assurance, TPS QA

## Abstract

**Background:**

Prior to the development of the IMRT and VMAT treatment techniques the quality assurance (QA) paradigm was one of routine linac QA supplemented by plan specific independent Monitor Unit checks and manual checks of data transfer. With the introduction of IMRT/VMAT treatment techniques the paradigm changed to include a patient specific measurement (PSQA) to ensure acceptable plan deliverability.

**Purpose:**

To inform upon whether QA procedures have improved to the point that measurement‐based PSQA is feasibly no longer required.

**Methods:**

The current status and future trajectory of QA procedures is assessed via a review of the literature pertaining to each of the three sub‐systems of deliverability, namely, treatment planning system (TPS) calculation accuracy, linac delivery performance, and data transfer between treatment planning system and linac.

**Results:**

The literature pertaining to 3D TPS dosimetry check systems used to assure TPS calculation accuracy is highly positive about such systems ability to provide at least equivalent level testing as measurement based PSQA with regards TPS calculation accuracy. The literature suggests that linac QA procedures have evolved since the advent of IMRT and methodology has been published for assessing the linac in its dynamic state used to deliver IMRT/VMAT. Data transfer QA has also evolved since the advent of IMRT and tests and procedures are now recommended in best practice guidance for both comprehensive data transfer QA programs and pre‐treatment checking and chart rounds. Additionally, the uptake of log file‐based QA methods provides a useful means of testing control point data transfer integrity.

**Conclusions:**

Contemporary sub‐system deliverability QA has evolved to the point where measurement based PSQA is not necessarily required.

**Abbreviations**
AAPM: American Association of Physicists in Medicine
ACR: American College of Radiology
ART: Adaptive radiotherapy
ASTRO: American Society for Radiation Oncology
CCC: Collapsed cone convolution
DLG: Dosimetric leaf gap
DVH: Dose volume histogram
EUD: Equivalent uniform dose
FMEA: Failure mode and effects analysis
H&N: Head and neck
ICRU: International commission on radiation units and measurements
IGRT: Image guided radiotherapy
IMRT: Intensity modulated radiotherapy
IPEM: Institute of physicists and engineers in medicine
IROC: Imaging and radiation oncology core
Linac: Linear accelerator
MIQC: Machine integrated quality control
MPC: Machine performance check
MPPG: Medical physics practice guideline
MU: Monitor unit
NCS: Netherlands Commission on radiation dosimetry
OAR: Organ at risk
PDD: Percentage depth dose
PSQA: Patient specific quality assurance
PTV: Planning target volume
QA: Quality assurance
ROC: Receiver operator characteristics
RPN: Risk‐priority‐number
SBRT: Stereotactic body radiotherapy
SGRT: Surface guided radiotherapy
SNC: Sun nuclear corporation
SPC: Statistical process control
SQA: Semi quantitative analysis
SRS: Stereotactic radio surgery
TG: Task group
TPS: Treatment planning system
UK: United Kingdom
VMAT: Volumetric modulated arc therapy
WHO: World Health Organisation

## 1 INTRODUCTION

One of the core challenges for radiation oncology medical physicists is ensuring that the dose distribution displayed by the treatment planning system (TPS) calculation is what is actually delivered to the patient by the treatment linear accelerator (linac). This challenge is ever‐present and evolves as new treatment technologies and techniques are introduced into clinical practice. Intensity modulated radiotherapy (IMRT) and volumetric modulated arc therapy (VMAT), including hypo‐fractionated Stereotactic treatments, were techniques developed in the 1990s^1^ and 2000s^2^ respectively that provided improved target dose conformality and reduced organ‐at‐risk (OAR) doses to patients compared to previous techniques. IMRT and VMAT involve changes to the beam aperture shape, beam intensity, and delivery angles during delivery and are considered to be significant improvements on previous techniques. In this article IMRT and VMAT will collectively be termed dynamic treatment techniques henceforth. Previous to the introduction of dynamic treatment techniques static field treatment techniques were standard and the deliverability quality assurance (QA) paradigm was based upon routine linac QA supplemented with plan specific independent Monitor Unit (MU) check of the TPS and manual data transfer checks between TPS and linac. When dynamic techniques were introduced, the difficulties in ensuring plan deliverability led to a paradigm shift in QA testing and the adoption of patient‐specific QA (PSQA) testing as industry standard.^3^ Deliverability PSQA testing typically involves the approved clinical treatment plan being delivered to a measurement device in the absence of the patient for assessment prior to the plan being used for clinical treatment. Measured doses are compared to the doses of the planned delivery with a pass/fail criteria applied to determine whether the treatment is to proceed as planned. Such a testing regimen is inefficient, since measurements are required for each individual treatment plan, and the ability to detect clinically significant treatment delivery inaccuracy has been questioned.^4^, ^5^, ^6^ In this study the proposition that a return to the original QA paradigm as has been argued by Siochi in a point/counterpoint,^7^ but with modernized methodology for dynamic techniques, is investigated via review of the literature. It is hypothesized that a return to the previous paradigm is feasible with contemporary QA methods so that deliverability PSQA measurements are not necessarily required.

The focus of this study is on PSQA techniques that assess deliverability for dynamic photon‐based treatments. Deliverability is defined here as how well the dose distribution delivered by the linac matches that simulated by the TPS not including patient variables. This definition aligns with that of McNiven et al.^8^ With this definition there is an assumption that the TPS will not produce a treatment plan requiring the linac to operate outside its normal expected range of operation. This assumption holds true for modern TPS. As such, deliverability, which measurement‐based PSQA is designed to assess, involves the three sub‐systems of TPS, linac and data transfer in between. It is noted that PSQA, for the purposes of deliverability as defined here, is a subset of all PSQA type activities. This study does not extend to the more generalized concept of PSQA that was recently presented by O'Daniel et al.,^9^ which included deliverability as defined here but also patient related variables. Such patient variables are monitored and controlled in radiotherapy practice using techniques such as image guided radiotherapy (IGRT), surface guided radiotherapy (SGRT), transit dosimetry and in vivo dosimetry. These practices, required for patient related variables, are outside the scope of this review, but are considered essential for safe and accurate radiotherapy treatment delivery.

In the study of O'Daniel et al.,^9^ failure–mode‐and effect analysis (FMEA) was applied to determine a list of radiotherapy failure modes that should be detected by their generalized definition of PSQA and which of these failure modes presented the greatest risk to treatment quality and safety. In their study, PSQA includes all treatment verification systems used to validate a specific patients treatment including direct measurements, secondary dose calculations, transit dosimetry, and in vivo dosimetry. The authors found that the highest ranking (RPN) failure modes were: TPS algorithm limitations, TPS commissioning errors, and patient weight variation, and noted that the TPS commissioning and algorithm limitations would ideally be caught during commissioning. O'Daniel et al., determined that with one exception that the top quartile of failure modes examined by severity suggested that standard quality management programs should detect these types of errors even without PSQA. These findings support the hypothesis of this study. If a comprehensive sub‐system QA program were implemented, then the FMEA approach of O'Daniel et al. would likely be useful for also assessing its effectiveness and whether there was a need for measurement based PSQA to cover any failure modes not detectable by the sub‐system QA program.

**FIGURE 1:**
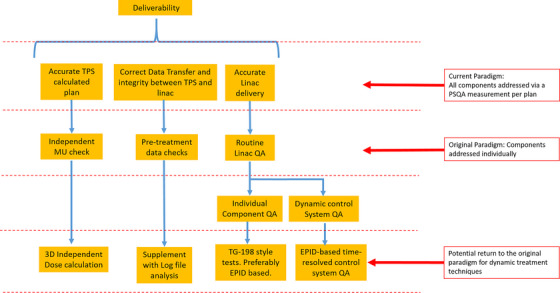
Schematic diagram of the deliverability components highlighting the three sub‐systems that are currently assured via PSQA, how these components were assured with the original QA paradigm and how the original paradigm could potentially be applied to modern dynamic treatment techniques.

Deliverability and associated QA paradigms are presented diagrammatically in Figure 1.

## 2 WEAKNESSES OF PSQA

As dynamic treatments became standard of care, PSQA became standard practice that was firmly entrenched when it became billable. With standard of care status, increased utilization of dynamic treatments quickly followed and PSQA became a core duty for medical physicists. PSQA methodologies were refined and improved. However, literature began to emerge highlighting the growing burden of PSQA on departmental resources and of inaccuracies and insensitivities of the methods. These bring into doubt the effectiveness of PSQA and its ability to detect the deliverability inaccuracies that is its purpose.

### 2.1 PSQA resource burden

Because of the large clinical uptake of dynamic treatment techniques the number of required PSQA measurements has increased proportionately. Aboloban et al.^10^ reported on a survey of United Kingdom (UK) radiotherapy departments in terms of dynamic treatment utilization and PSQA patterns. The survey was initially performed in 2012 and repeated in 2014 with changes in practice in this period reported upon. Reducing the PSQA burden was common feedback from respondents with 42% wanting to introduce EPID‐based methods, 48% wanting to introduce software‐based methods and 25% wanting to stop performing PSQA at all. The authors concluded that PSQA was still a significant burden on radiotherapy departments and that it was a barrier to increased dynamic treatment utilization.

Barber et al.^11^ reported on a 2015 survey of Australian and New Zealand departments on the equipment, policies, and procedures for the commissioning and QA of dynamic treatment techniques. With regards resource burden the authors concluded that PSQA remained a significant burden on medical physics workload, but that moving away from performing a measurement for each plan was contentious.

A very recent survey of UK centers by Tassano‐Smith et al.,^12^ indicated highly inconsistent PSQA practices in the UK. The proportion of plans for which measurement‐based PSQA was performed ranged from 1% to 100%, but in the majority, relatively small percentages of plans were measured. These results could indicate declining measurement‐based QA rates, but it is unclear if this is limited to the UK context. However, 49% of UK centers still use measurement‐based QA when a new technique or other change to the planning process is introduced, 21% perform a measurement for complex plans and 19% when MU or MU/Gy limits are breached. 30% perform a measurement on randomly sampled plans.

### 2.2 PSQA inaccuracies and insensitivities

Three seminal studies have called into question the effectiveness of measurement based PSQA.^4^, ^5^, ^6^ Ford et al.^4^ investigated the effectiveness of commonly used quality control measures in detecting clinically significant errors. PSQA was found to detect only 1.4% of the error scenarios reported and the authors suggested that pre‐treatment measurement based PSQA is often considered indispensable but offers very little additional value in terms of error detection.

Kry et al.^5^ compared PSQA sensitivity and specificity against imaging and radiation oncology core (IROC) phantom‐based dosimetry audit results. In terms of ability to predict a failed phantom result, overall PSQA sensitivity ranged between 2% and 18% with different PSQA methods ranging between 3% and 54%. The authors concluded that PSQA was not a reasonable replacement for a credentialing phantom and that there was high inconsistency in PSQA performance.

More recently, Lehmann et al.^6^ performed a study whereby a stereotactic spine treatment plan was successively copied and modified via the introduction of deliberate simulated errors ranging from sub‐clinical to significant in their effect on the delivered dose distribution. Blinded to which plans had the errors, 17 centers performed PSQA on each of 12 plans and reported which they would classify as pass or fail. It was found that 17 plans across 7 institutions passed plans with errors that could be expected to result in >5% increase in spinal cord dose to the patient. Six plans from four institutions passed plans expected to result in spinal cord dose >10% above expected. The authors concluded that the inclusion of deliberate errors in auditing could be useful for highlighting deficiencies in local centers QA procedures.

Besides the three seminal studies presented on PSQA ineffectiveness numerous other studies have highlighted inaccuracies, insensitivities, and weaknesses in PSQA processes. These include insensitivities to deliberate introduced errors,^13^, ^14^, ^15^, ^16^, ^17^, ^18^ insensitivity of field‐by‐field PSQA,^19^, ^20^, ^21^ and weaknesses of the commonly used Gamma metric.^15^, ^20^, ^22^, ^23^, ^24^, ^25^, ^26^, ^27^ These studies call into question the effectiveness of measurement based PSQA in its ability to detect clinically meaningful errors. This likely contributes as to why 36.2% of respondents of the Aboloban et al. survey^10^ and 23% of the Tassano–Smith^12^ study indicated that they never modified a plan based on PSQA results. However, a recent study by Dunn et al.^28^ indicated that PSQA could be sensitive to clinically relevant errors if appropriate tolerances were set. These findings were in alignment of those of Xia et al.^29^ and provide a counter argument that with appropriate tolerances set, PSQA can be sensitive to clinically meaningful errors.

### 2.3 Alternatives to measurement based PSQA for every plan

With the weaknesses inherent to measurement based PSQA and the emergence of online adaptive treatments whereby a patient is replanned and treated while on the treatment couch each day and hence it isn't possible to do a pre‐treatment measurement for each new plan there is a growing need to revise the PSQA paradigm. There are alternate potential pathways forward. The Netherlands Commission on Radiation Dosimetry (NCS) suggested that PSQA measurements need not be performed for every treatment plan once a significant history of PSQA had been established for the patient cohort.^30^, ^31^ Another option, which has been the subject of many recent studies is the concept of using methods to predict which plans will fail PSQA in an effort to reduce the number of plans requiring PSQA measurement. Predictive methods have been presented based upon plan complexity metrics^8^, ^32^, ^33^, ^34^, ^35^, ^36^, ^37^, ^38^, ^39^, ^40^, ^41^, ^42^ with the reader referred to the publication of Antoine et al.^43^ for a more comprehensive systematic review. More recently predictive methods based upon both secondary dose calculation^44^, ^45^, ^46^, ^47^ and Machine Learning and Artificial Intelligence techniques^48^, ^49^, ^50^, ^51^, ^52^, ^53^, ^54^, ^55^, ^56^, ^57^, ^58^, ^59^ have been reported.

A potential alternative, which is the focus of this study, is whether a return to the original QA paradigm is feasible considering advances in QA techniques. Treatment plan check systems have evolved from the simple MU checkers of the past and are now sophisticated independent dose engines that can now assess the 3D dose distribution using clinically meaningful metrics. The uptake of log file analysis, where log files are generated by the linac and include information as to what the linac believed it had delivered, is a useful source of information particularly for assessing whether the correct plan had been delivered and whether it had retained data integrity in the transfer from TPS to linac. In terms of linac QA, there is likely still a role for static type testing and best practice recommendations such as those from the American Association of Physicists in Medicine (AAPM) Task Group (TG) 142,^60^ TG‐198,^61^ Medical Physics Practice Guideline (MPPG) 8.b.^62^ and from the Institute of Physics and Engineering in Medicine (IPEM) report 81 2nd ed^63^ are still relevant. Modern linac QA techniques, often EPID‐based, can provide a means of performing such tests in a highly efficient manner.^64^, ^65^, ^66^, ^67^, ^68^, ^69^ However, an insufficient aspect of linac QA has been that of dynamic control system QA whereby the dynamic systems are tested in the dynamic state for which they will be used for dynamic treatment delivery. Attempts have been made to address this shortfall.^70^, ^71^, ^72^ Log file analysis has been used for the purpose of linac dynamic control system QA. This is cautioned against due to the lack of independence of log files from the systems under investigation^73^ and because of the demonstrated insensitivity to linac, particularly MLC, failure modes.^74^, ^75^, ^76^, ^77^ A potentially viable method is time resolved EPID measurement, which has the features of being both independent of the system under investigation, even if not independent of the linac itself and being time resolved so that the dynamic treatment can be assessed with a dynamic measurement. The concept has been demonstrated by Zwan et al.^78^, ^79^ and Lim et al.^76^

The following sections will go into the detail from the literature as to the suitability of contemporary sub‐system QA for assuring dynamic treatment deliverability in the absence of a PSQA measurement. It should be noted that this study was not commissioned to provide guidance and the conclusions drawn are those of the author and the reader may draw different conclusions from the literature presented. The role of this review is primarily to provide the reader with an overview of the relevant literature, current at the time of writing, to help them assess for themselves the value of their PSQA program.

## 3 CONTEMPORARY QA METHODS RELEVANT TO DELIVERABILITY SUB‐SYSTEMS: A LITERATURE REVIEW

### 3.1 PS dosimetry checking systems

Evidence exists that the TPS can be a significant source of deliverability inaccuracy.^9^ The PSQA FMEA study of O'Daniel et al.^9^ found that TPS failure modes were among the highest risk failures and that these failures occurred at an institutional level rather than at the patient level. Such institutional fails should be detected during the TPS commissioning process, guidance on which is provided in AAPM MPPG 5.b.^80^ However, despite the systemic nature of these errors, they are still prevalent and independent dose recalculation systems have been found to be well suited for detecting at least some of the relevant inaccuracies.^81^, ^82^ Such per‐patient TPS dosimetry checks were, in the form of independent MU checks, utilized prior to the invention of IMRT for conformal beam treatments and a TPS dosimetry check per patient is still recommended in AAPM TG‐219.^83^

There now exists multiple sophisticated and independent 3D TPS dosimetry checking systems. Literature on the performance of these systems have been the subject of many studies with relevant publications summarized in Tables 1, 2, and 3. AAPM Task Group 219 was commissioned to provide best practice recommendations for the implementation and use of such systems.^83^

**TABLE 1:** Summary of the literature evaluating Mobius 3D (CCC algorithm) as TPS dosimetry check tools.

Publication	Contribution	Key findings/Conclusions
Nelson et al., 2014^85^	Compared Mobius3D in simple phantom geometries to measurement. Compared Mobius 3D to TPS and to PSQA type measurements for dynamic treatment plans.	PDD differences for MLC defined fields within 2.28%, in‐field profile differences within 0.61%, 98.1 ± 5.3% gamma pass rate compared to TPS at 3%/3mm criteria. Suggested that based on the preliminary results of the study that Mobius 3D should be able to calculate dose adequately as a verification tool of the TPS.
Fontenot et al., 2014^87^	Evaluated Mobius 3D for prostate, lung, and H&N PSQA via comparison to TPS and measurement using Gamma and DVH analysis	Suggested comparable accuracy of Mobius 3D to TPS and identified several plans that exceeded DVH limits.
Clemente‐Gutierrez et al., 2015^86^	Assessed Mobius 3D in simple phantom geometries compared to measurement. Also assessed Mobius 3D with 2D gamma analysis comparison to measurement for TG‐119^96^ test plans and real VMAT plans.	Output factor agreement within 1%, PDD agreement within 2.7%, in–field dose profile agreement within 2.8% and penumbra within 1.5 mm distance to agreement. Mobius 3D was successfully validated for clinical use.
Au et al., 2017^94^	Sensitivity of Mobius 3D was evaluated via introduction of errors into test plans and compared to that of the ArcCheck PSQA device.	Mobius 3D was found to have comparable error detectability to ArcCheck and was able to detect 2° collimator rotation errors, 1 mm MLC bank offsets and 10 mm jaw offsets.
Jolly et al., 2017^95^	Presented Mobius 3D results for 1000 cases to statistically set tolerances for 2 TPS systems and the lung treatment site.	Statistical tolerances were determined for Gamma pass rate (3%/3mm) and mean target dose
McDonald et al., 2017^88^	Mobius 3D was evaluated against point dose measurement in phantom for 17 IMRT plans. Results were compared to the point dose agreement with the TPS.	The dose calculation accuracy and independence of Mobius 3D is sufficient to provide a rigorous second check of a modern TPS.
Hillman et al., 2018^93^	Mobius 3D was evaluated for SRS/SBRT PSQA using 3D gamma analysis. The Mobius 3D beam model was refined for greater small field accuracy required for these treatment types.	If the Mobius 3D beam model is refined for small fields then the system could become a premier SRS/SBRT QA tool.
Kodama et al., 2019^89^	Evaluated Mobius 3D for Tomotherapy PSQA via comparison to measurement in phantom and via 3D gamma passing rates for prostate, H&N and Oesophagus tumours.	Mobius 3D needs to be locally commissioned and treatment site specific tolerances set.
Ce Han et al., 2020^90^	Assessed Mobius 3D vs ArcCheck for H&N, chest and abdominal VMAT PSQA using DVH and gamma analysis.	Differences were observed between ArcCheck and Mobius 3D and the authors suggested that care must be taken when considering replacement of measurement‐based IMRT/VMAT QA.
Basavatia et al., 2021^91^	Mobius 3D was evaluated against measurement based PSQA for IMRT using gamma analysis.	Concordance was observed between Mobius 3D and conventional PSQA methods and that Mobius was a suitable alternative to measurement based PSQA that could improve efficiency and workflows.
Hasse et al., 2021^44^	ROC analysis and Machine learning were applied to Mobius 3D results in an attempt to determine which plans required measurement based PSQA.	It was deemed possible to identify plans that are more likely to fail ion chamber based PSQA before delivery, which could free up significant resources.
Cavalli et al., 2024^92^	Mobius 3D was evaluated as a PSQA tool for SRS HyperArc treatments using gamma and DVH analysis.	Mobius 3D has better error detectability than conventional measurement based PSQA.

**TABLE 2:** Summary of the literature evaluating SciMoCa (Monte Carlo algorithm) as TPS dosimetry check tools.

Publication	Contribution	Key findings/Conclusions
Hoffman et al., 2018^100^	Validated Acuros TPS calculation via comparison to SciMoCa for 3DCRT lung, IMRT lung, H&N VMAT, Cervix IMRT, and rectum VMAT via gamma and DVH analysis.	Acuros and SciMoCa calculated dose distributions were identical within implementation detail and fundamental cross section data uncertainties.
Milder et al., 2020^102^	Evaluated SciMoCa for CyberKnife PSQA via comparison to simple phantom measurements and comparison to the Octavius PSQA system via SPC tolerance setting.	For output factors the maximum recorded difference was 3.2% with 99% of measurements agreeing to within 2%. PDD and dose profiles demonstrated average gamma pass rates at > 99% and 88% respectively at 2%/0.5 mm. In terms of clinical plans, SciMoca indicated a 98% pass rate at 2%/1mm criteria. Statistical process control (SPC) analysis detected six plans outside tolerance levels in the Octavius measurements, all of which were attributed to measurement setup while SciMoCa indicated five plans outside of tolerance, all of which, under investigation were attributed to differences in the TPS and SciMoCa algorithms. SciMoCa was considered validated and its performance sufficient to replace measurement based PSQA.
Piffer et al., 2021^97^	Evaluated SciMoCa for two TPS (Monaco and Pinnacle) systems for 50 VMAT plans via comparison to ArcCheck measurement using point dose and gamma analysis.	For point doses, SciMoCa and TPS average agreement with measurement was within 0.7% and –0.2% respectively. Direct comparison between SciMoCa and the two TPS revealed point dose differences that were statistically insignificant and 98% and 99% gamma pass rates were recorded respectively. SciMoCa based PSQA was considered to be clinically viable, reliable that potentially allows for significant time saving.
Szeverinski et al., 2021^98^	SciMoCa was evaluated for prostate VMAT PSQA for the Monaco TPS using gamma and DVH analysis on 30 treatment plans. SciMoCa sensitivity to MLC shift was also assessed.	Comparison to ion chamber results were clinically acceptable. Gamma pass rates ranged between 96.10% and 100.0%. SciMoCa calculations of Monaco TPS plans are in excellent agreement to each other.
Adamson et al., 2023^99^	Evaluated SciMoCa for SRS PSQA purposes and presented the commissioning and implementation process.	99.2% pass rate observed for a 2%/1mm criteria. In comparison to OSL and scintillator measurements in anthropomorphic phantom a –1.9% mean difference was observed. For 10 patient cases agreement between SciMoCa and TPS was observed at –0.8%, –1.3%, and –0.5% for PTV mean dose, D95% and D1%, respectively, which via AAPM TG‐218^3^ methodology corresponded to passing rate action limits of ±5.2%, ±6.4%, and ±6.3% respectively. Beam modelling, validation, and tolerances presented could be used as a benchmark for future SciMoCa users for single isocenter multi target SRS.
Kowatsch et al., 2023^101^	Aimed to develop clinically meaningful tolerance levels for SciMoca. utilizing ROC analysis of results for plans with deliberately introduced errors.190 un‐modified clinical plans generated with the Monaco TPS were assessed along with a further 190 plans for which deliberate dose errors in the range of 1.5%–2.5% had been introduced. Receiver‐operator‐characteristics (ROC) analysis was performed for a range of gamma criteria and optimum action levels were derived via Youden's J statistics.	SciMoCa is highly efficient at catching errors in the treatment planning process. A dose difference criterion of 2% and distance to agreement of 1 mm was considered the appropriate tolerance level for a Monte Carlo based TPS. SciMoCa is highly efficient at catching errors in the treatment planning process.
Ruggieri et al., 2025^103^	Evaluated SciMoca to provide QA of plan‐of‐the‐day Adaptive RT for an MRI‐linac system.	Gamma pass rates for PDDs and transverse dose profiles against commissioning measurements were measured to be 99.1% and 99.3% for TPS and SciMoCa respectively using a 2%/1mm criteria. Output factors were within 1% for both systems down to a 1 × 1 cm field size. For the lung phantom PDD, SciMoCa was found to show reduced insensitivity to the variations observed in the TPS. In the clinical plan SciMoCa indicated a slightly higher V95% for pancreas and lung treatments and slightly higher target D2% for all sites. In comparison to in‐phantom dose measurements SciMoCa showed an average 1% increase in pass rate compared to TPS. SciMoCa was considered to have equivalent beam model quality to the Monaco TPS making SciMoCa useful for daily validation of Monaco based online approval decisions.

**TABLE 3:** Summary of the literature evaluating SNC DoseCheck (CCC algorithm) and RadCalc (CCC and Monte Carlo algorithms) as TPS dosimetry check tools.

Publication	Contribution	Key findings/Conclusions
**SNC Dose Check**		
Bismak et al., 2022^104^	Evaluated the performance of per‐linac custom beam models for Dose Check using TG‐119^96^ and clinical IMRT/VMAT plans compared to the AAA algorithm ad film measurement.	DoseCheck shows agreement within TG‐119 confidence limits to film measurement and in comparison to AAA calculated dose distributions. TG‐119 plans can be useful for assessing DoseCheck models and for clinical baseline setting.
Baltz et al., 2023^46^	Assessed DoseCheck as a tool for screening to determine whether SRS and SBRT plans required a measurement based PSQA assessment.	A strong correlation was observed between DoseCheck and SNC SRS MapCheck measurement suggesting that the same actin limits could be applied to both and that DoseCheck could successfully be used to predict SRS MapCheck results and hence be used for screening.
**RadCalc CCC and Monte Carlo**		
Richmond et al., 2023^105^	Evaluated the RadCalc CCC algorithm against measured PDD, beam profiles, heterogeneities, absolute dose, and IMRT/VMAT test plans.	PDDs within 0.5% beyond 2 cm depth. Beam profiles within 2% in the central 80% region and out of field dose underestimated by up to 3%. Dose comparisons in heterogeneities within 3.5% with largest differences at interfaces. Static IMRT fluences with 2%/2mm and complex VMTA deliveries with 3%/2mm. The primary collimator is not modelled in RadCalc leading to overestimation of dose in the corners of large fields.
Sceni et al., 2023^106^	RadCalc Monte Carlo models were tuned for the DLG parameter to provide the best agreement with measurement based PSQA data.	After model tuning, RadCalc Monet Carlo was in good agreement with TPS simulations and provides a better representation of plan doses in lung cases. The inclusion of inhomogeneity in RadCalc Monte Carlo is an improvement over homogenous phantom assessments, which measurement based PSQA typically utilize.
Mastrella et al., 2025^107^	Evaluated RadCalc Monte Carlo and RadCalc CCC for clinical use with the Pinnacle TPS. Assessment was performed for in‐phantom, along with patient datasets and against ion chamber measurement.	In phantom and clinical plans (H&N and SBRT) results showed RadCalc‐TPS mean gamma pass rates >98%. RadCalc Monte Carlo performed better for H&N but CCC better for other sites. RadCalc produced very consistent results with the Pinnacle TPS and could successfully be used for PSQA. Averaging results from both RadCalc algorithms reduced uncertainties.

International Commission on Radiation Units and Measurements (ICRU) report 83 suggests that for the purpose of PSQA that as an alternative to a measured assessment of the dose distribution accuracy that it is acceptable to use an independent absorbed‐dose calculation. Such a calculation is required to both be a three‐dimensional assessment and that it is at least as accurate as the TPS calculation being assessed.^84^ AAPM TG‐219^83^ provided a list of commercially available independent TPS dosimetry check systems and described the underlying algorithms in detail. The report summarizes the accuracy of the algorithms in different clinical scenarios and finally discusses methods for the commissioning and safe implementation of these systems.

The Varian Mobius 3D system and the SciMoCa Monte Carlo based system are the 3D TPS dosimetry check systems most published upon in the literature. The other systems reported on in this review are the Sun Nuclear (SNC) (Sun Nuclear Corporation, Melbourne, Florida, USA), DoseCheck and RadCalc (Lifeline Software Inc, Tyler, Texas, USA), Collapsed Cone Convolution (CCC), and Monte Carlo systems. It is acknowledged that there are other systems commercially available and new systems will potentially also come on to the market into the future, but for the purposes of brevity this review has been limited to four systems that have been published upon.

#### 3.1.1 Varian Mobius 3D

The Varian Mobius 3D system utilizes a CCC algorithm and has been extensively evaluated in the literature with publications summarized in Table 1. Studies have been performed assessing performance of Mobius 3D against basic commissioning measurements^85^, ^86^ and against TPS calculated and measured dose distributions for both IMRT and VMAT treatment techniques.^44^, ^85^, ^86^, ^87^, ^88^, ^89^, ^90^, ^91^, ^92^ Performance has been assessed for a variety of treatment sites including H&N,^87^, ^89^, ^90^ prostate^87^, ^89^ lung,^87^ chest,^90^ abdominal^90^ and cranial stereotactic radiosurgery (SRS)^92^, ^93^ using 2D and 3D gamma pass rates,^85^, ^88^, ^89^, ^91^, ^92^, ^93^ and dose‐volume‐histogram (DVH)^86^, ^87^, ^88^, ^90^, ^91^, ^92^ assessment metrics. The sensitivity of Mobius to deliberately introduced errors has also been assessed^92^, ^94^ and studies have been performed to indicate suitable tolerances.^89^, ^95^ Different TPS were assessed and while most studies related to conventional linacs, one study was specific to the Tomotherapy system.^89^ All studies were favorable of the performance of Mobius3D with conclusions reached that Mobius3D's accuracy was sufficient to provide a rigorous second‐check of a modern TPS, that Mobius 3D could be a suitable alternative to conventional measurement based QA methods for SRS Hyperarc treatments and that Mobius's combined detectability is comparable to that of ArcCheck and higher than the 3DVH system. One study applied machine learning to Mobius 3D results in comparison to ion chamber point doses in an effort to predict which plans may not need an ion chamber measurement for verification.^44^ Results indicated that fewer than 25% of all plans required a physical dose measurement if a Mobius 3D calculation had been performed.

#### 3.1.2 SciMoCa

SciMoCa (Scientific RT, Munich, Germany) is an independent 3D TPS dosimetry checking system based upon Monte Carlo calculation that has been extensively evaluated in the literature. Publications are summarized in Table 2. SciMoc has been evaluated relative to measured data^97^, ^98^, ^99^ for conventional linacs with authors universally positive about SciMoCa's performance as a PSQA tool. SciMoCa has also been evaluated in comparison to TPS calculated dose distributions^97^, ^100^ for the Varian Acuros XB algorithm, Monaco (Elekta, Stockholm, Sweden) and Pinnacle (Philips Radiation Oncology Systems Fitchburg, USA). It was concluded that SciMoCa dose distributions were in outstanding agreement with Acuros and that with regards Monaco and Pinnacle that SciMoCa was clinically viable as a PSQA tool, is reliable and allows for potentially significant time saving. A sensitivity study utilizing deliberately introduced errors has been performed that also provided tolerance recommendations.^101^ The authors concluded that SciMoCa was highly efficient at detecting errors in the treatment planning process. SciMoCa has also been evaluated for the Cyberknife system (Accuray, Sunnyvale, CA, USA)^102^ and for a 1.5 Tesla MRI‐linac system^103^ with authors concluding that SciMoCa had been successfully commissioned and validated to replace PSQA measurements for the CyberKnife system and that for the MRI‐linac that SciMoCa had equivalent beam‐model quality to Monaco and that SciMoCa was hence a useful tool for plan verification purposes.

#### 3.1.3 DoseCheck and RadCalc

The SNC DoseCheck utilizes a CCC algorithm and the RadCalc 3D dosimetry check system is available with either CCC or Monte Carlo algorithms. The performance of these systems has been reported upon with relevant studies summarized in Table 3. Two studies have evaluated DoseCheck^46^, ^104^ and three studies have evaluated RadCalc.^105^, ^106^, ^107^ For DoseCheck, evaluation was performed using TG‐119^96^ and clinical plans via comparison to AAA algorithm calculation and to measurement. Authors concluded that the DoseCheck algorithm showed agreement well within TG‐119 confidence limits for film measurement and redundant dose calculation comparison with AAA and that DoseCheck could successfully be used as a measurement screening tool for the treatment sites investigated.

With regard to RadCalc, the CCC algorithm has been evaluated against simple measured data and complex IMRT and VMAT test fields.^105^ In this study it was discovered that RadCalc significantly overestimated in the corners of large fields due to the primary collimator not being accounted for in the RadCalc model, but generally good agreement was observed overall. For RadCalc Monte Carlo the process to best tune the model has been presented with good observed agreement with TPS being observed post tuning and the benefits of calculation on the patients CT dataset were recognized.^106^ In one study the performance of RadCalc CCC and Monte Carlo were both evaluated in phantom and on clinical datasets compared to measurement.^107^ It was observed that RadCalc Monte Carlo reported better agreement for H&N, whereas CCC reported higher for other sites. It was also reported that measurements may underestimate calculation discrepancies due to issue inhomogeneities. The authors concluded that use of RadCalc could reduce the measurement based PSQA burden and that averaging the RadCalc algorithms could reduce calculation uncertainties.

#### 3.1.4 Summary and critical review of the literature

Multiple 3D TPS dosimetry checks systems utilizing both CCC and Monte Carlo algorithms are commercially available and have been evaluated in the literature, which is highly positive as to their performance. Systems have been assessed across a wide range of treatment sites, across different TPS and differing delivery systems. Clinically acceptable performance of multiple different systems has been demonstrated against measurements ranging from simple commissioning type tests (PDDs, profiles etc) up to complex PSQA type measurement. Multiple systems have also demonstrated acceptable performance in comparison to TPS calculation using both DVH and Gamma metrics. Sensitivity to simulated errors has also been demonstrated. Studies have also provided clinically meaningful tolerances based upon both statistical methods and agreement to measurement. Best practice guidance also exists that both allows TPS dosimetry check systems in place of PSQA measurement and provides recommendation as to their effective use. As such, the body of literature suggests that in conjunction with a well commissioned TPS that utilization of 3D TPS dosimetry check tools with appropriate tolerances can provide effective TPS calculation QA meaning that measurement based PSQA is not necessarily required for the TPS sub‐system. All that is potentially required is an FMEA type study that ideally identifies all of the significant TPS failure modes and demonstrated detectability of 3D TPS dosimetry check systems to each.

### 3.2 Data transfer QA

The role in assuring integrity of data transfer is an aspect of measurement‐based deliverability PSQA that is often not well understood. Delivering a treatment plan to a measurement device and comparing the measured results against the clinically approved plan tests several data transfer parameters that are difficult to assess by traditional pre‐treatment and chart round checks. If measurement‐based deliverability PSQA were to no longer be performed then alternate methods would be required for assuring data transfer integrity.

Best practice guidance on data transfer QA is provided in AAPM TG‐201.^108^ In TG‐201 the authors justify the need for data transfer QA based upon the findings of the World‐Health‐Organisation (WHO) that reported that data transfer errors accounted for 38% of near misses and 9% of incidents.^109^ The authors also point to the significant radiotherapy injuries and death presented by the New York Times, which were attributable to data transfer type faults.^110^, ^111^, ^112^ In terms of existing guidance TG‐201 suggests that while many AAPM best practice guidelines refer to the need for comprehensive data transfer QA, many are short on specifics either referring to the matter in general^113^, ^114^ or simply recommending end‐to‐end testing.^60^, ^80^, ^115^ Both the American Society for Radiation Oncology (ASTRO) and the American College of Radiology (ACR) also generally recommend end‐to‐end testing.^116^, ^117^, ^118^, ^119^, ^120^, ^121^ For a more detailed summary of best practice guidance with regards data transfer QA the reader is referred to AAPM TG‐201.^108^

AAPM TG‐201 suggests that the risks to clinical data include loss of data integrity (i.e. data corruption), multiple data copies that differ from each other (i.e. wrong data used) and data caches (i.e. non‐current data). The study of Zhang et al.^122^ also highlighted the risk of cyber‐attack and presented a method to mitigate this risk, which was successful under simulated conditions.

Data transfer risks differ between multi‐vendor environments, whereby each sub‐system typically stores a copy of the data, and single vendor environments whereby data is typically stored in a central database but may be read by sub‐systems at different times.^108^ The study of Donahue et al.^123^ suggested that multi‐vendor environments were particularly susceptible to data transfer errors. Over an 8‐month period 2774 plans were evaluated from which 19.7% were found to have had at least one failure detected. Most errors detected involved manual steps in the transfer process and the authors concluded that checking such manual steps be the priority.

To mitigate data transfer risks, TG‐201 recommends a data transfer QA program that includes acceptance testing, commissioning, end‐to‐end testing, and annual QA, and when a sub‐system upgrade occurs, acceptance testing must be repeated. This regimen should ideally include computer‐based check systems and would be supplemented by the checks performed pre‐treatment by radiation therapists and those by medical physicists during chart rounds as part of the quality control system for each individual patient. Sub‐system fault recovery is also understood to be a significant point of risk for data error. Discussion and recommendations regarding fault recovery to mitigate the risk are provided in AAPM TG‐314.^124^

According to TG‐201, the data transfer quality control process has two distinct elements: integrity of the data between sub‐systems and the logical consistency of the data after transfer. For the former, data needs to be checked post versus pre‐transfer and vendors now usually include checksums in their systems to aid this process. The remainder is checked manually or using validated software solutions. For the logical consistency of data, review by a user is still the only available method.^108^ As such, pre‐treatment checks and chart rounds continue to play an important role in assuring accurate plan delivery to the patient, although these can and should be assisted by automated software solutions with appropriate validation and oversight.^108^, ^125^ Guidance on treatment plan and chart checks is provided in AAPM TG‐275^125^ and it is noted that a check of technical parameters (e.g. data transfer integrity) is the first aspect of plan/chart reviews that is listed.

Data transfer checks that should be considered in the individual patient quality control program are discussed in TG‐201.^108^ Most of the checks listed can be performed manually as part of a therapist pre‐treatment check or physicist chart round. The task that is estimated to take the longest time is the check of control point data. This requirement came about with the introduction of IMRT and can be considered to be covered with a PSQA deliverability measurement. If PSQA deliverability measurements were no longer performed, then an alternate method would be required to check this aspect of data transfer. It is contended that machine log files could provide a data source for convenient, but thorough control point data transfer testing. Several authors have attested to the sensitivity of log files via experiments whereby machine errors have been simulated via modification of the treatment plan.^28^, ^126^, ^127^, ^128^, ^129^, ^130^ Although subsequent publications call into doubt whether log files are sensitive to the actual linac failure modes,^74^, ^75^, ^76^, ^77^ the studies could be interpreted to suggest that log files are sensitive to small, but significant changes in control points as could occur with a data transfer error or wrong treatment plan version. Log file analysis used for this purpose could likely in combination with manual or automated pre‐treatment checks meet the data transfer quality control requirements of TG‐201 and hence a pre‐treatment PSQA deliverability measurement would no longer be required for this purpose.

#### 3.2.1 Summary and critical review of the literature

The significant body of literature on contemporary data transfer QA suggests that data transfer integrity for dynamic treatment techniques can likely be assured without the need for measurement based PSQA. Best practice guidance exists outlining the requirements of a data transfer QA program. Manual checks are still recommended to be performed by both Medical Physicists and Radiation Therapists following best practice recommendations. Such checks can be augmented by automated checks systems, built‐in machine checks sums, and potentially log file analysis. Best practice guidance is also available for fault recovery procedures and literature exists for mitigating the risk of cyber attack. The body of literature would appear comprehensive and if followed should mitigate the need for measurement based PSQA for assuring data integrity. This could be proven with an FMEA type study that ideally identifies all of the significant data transfer failure modes and then demonstrated detectability of a data transfer QA program to each failure mode would negate the need for PSQA for assuring data transfer integrity.

### 3.3 Linac QA for dynamic delivery techniques

#### 3.3.1 Clinical impact of treatment delivery errors

The justification for dynamic treatment technique linac QA testing is based upon several studies in the literature which have determined the clinical significance of potential linac errors in the delivery of dynamic treatment plans. These studies typically modified clinical treatment plans with simulated changes to various linac components. Plan quality metrics such as DVH statistics were then used to determine clinical significance. Publications in the 2000's generally related to IMRT and those in the 2010's generally related to VMAT. From the mid‐2010's articles related to SBRT/SRS began to be published. The literature for each of these dynamic treatment types is now presented and the publications are summarized in Tables 4, 5, and 6.

**TABLE 4:** Summary of the literature pertaining to the clinical impact of dynamic treatment delivery errors for the IMRT treatment technique.

Publication	Contribution	Key findings/Conclusions
Low et al., 1997^143^	Investigated the impact of collimator and gantry angle errors on IMRT pelvis plans for different beam energies.	Gantry angle error of 2° for the 6 MV beam resulted in dose error of greater than 40%.
LoSasso et al., 1998^1^	Investigated the impact of small‐field scatter factor, collimator transmission, and MLC leaf tip errors on IMRT prostate plans.	If not accounted for the errors investigated could result in errors in the dose to the prostate between 5% and 20%.
Xing et al., 2000^144^	Assessed the dosimetric impact of gantry and collimator angle errors on spine and H&N IMRT plans.	5° change in gantry angle of one of nine IMRT beams resulted in a decrease of 1.5% in minimum target dose and 5.1% in spinal cord maximum dose.
Cadman et al., 2002^22^	Investigated the impact of incorrect MLC leaf gap on a H&N dosimetry audit phantom.	A maximum underestimate of calculated dose of 12% was observed for no leaf gap correction and that when a 1.4 mm leaf gap correction was applied, agreement between measurement and calculation was within 5%.
Litzenberg et al., 2002^140^	Simple and complex IMRT fields were sequenced using different leaf speeds and tolerance settings to identify limitations of the linac control systems ability to provide an accurate delivery.	Limiting the leaf sequencer for maximum leaf velocity, leaf position tolerance, communications delay, and collision avoidance resulted in deliveries which did not require beam interrupts or dose rate modulations
Xia et al., 2002^141^	Assessed the linacs ability to accurately deliver IMRT fields with small MU and different dose rates.	Delivery inaccuracy was observed for small MU and/or high dose rate.
Parsai et al., 2003^134^	The clinical impact of random and systematic MLC and diaphragm errors was assessed on a meningioma plan.	Random incorrect positioning of the MLC or the backup diaphragm of magnitude 1.5 mm could cause dose discrepancies above 5%, while systematic errors of only 0.5 mm produce more significant dosimetric deviation. 1 mm random errors of both MLC and diaphragm can lead to a 5% error in prescribed dose. However, for error in each system alone a 1.5 mm magnitude error was required for a 5% dose error. Systematic errors as small as 0.5 mm resulted in insignificant dosimetric deviations.
Stell et al., 2004^142^	Evaluated the impact of MU, leaf motion errors, and dose rate on step‐and‐shoot IMRT plans using log file analysis.	Segment MU errors were observed to increase with dose rate. Total absolute MU error was observed to increase with the number of plan segments, exacerbated for higher dose rates. These errors were not found to be clinically significant. At 600 MU/min, between 5% and 23% of the plan MUs were delivered during MLC leaf motion with 1 mm error.
Luo et al., 2006^135^	Recalculated dose distributions based upon log file data using Monte Carlo for IMRT plans.	A linear correlation between target dose error and average MLC position error. Average MLC position error of 0.2 mm can result in target dose error of about 1%.
Lee et al., 2007^131^	Investigated the impact of MLC dosimetric leaf gap for small beam fields on an IMRT test plan.	Maximum dose difference for in‐PTV, outside‐PTV and in an OAR were reduced from 22.3% to 5.5 %, 20.2% to 8.5%, and 35.2% to 6.3% respectively between a 0 and 2 mm leaf gap setting.
Wasbo et al., 2008^132^	Investigated the dosimetric impact of IMRT treatment on nominally matched linacs with differing MLC transmission and dosimetric leaf gap.	Dosimetric differences were unacceptable for 1 out of 4 linacs investigated.
Mu et al., 2008^136^	Investigated the clinical significance of random and systematic MLC errors on IMRT H&N plans of differing complexity.	Random MLC errors were insignificant up to 2 mm for simple and complex plans. For a 1mm systematic error average changes in D95% were 4% for simple plans and 8% for complex plans and 9% and 13% for average parotid dose for simple and complex, respectively.
Rangel and Dunscombe, 2009^137^	Investigated the clinical significance of random and systematic MLC errors on IMRT prostate and H&N plans	For every 1 mm of systematic shift in MLC position the difference in the reference equivalent uniform dose was approximately 2.7% for a typical IMRT prostate plan and 5.7% for a typical IMRT H&N plan. Systematic MLC errors of 0.3 mm could lead to a 2% change in target EUD and 2 Gy for OARs. Random MLC errors were insignificant up to 2 mm for both target coverage and OARs.
Bai et al., 2013^138^	Assessed the impact of MLC leaf position and collimator and gantry rotation angle errors on H&N IMRT plans.	MLC field size errors were significant down to 0.5 mm. At 0.5 mm systematic MLC shift the mean dose for the parotids increased by up to 4.6%. The impact of field offset errors and random errors was insignificant. Collimator and gantry angle errors were insignificant up to 0.5°.
Agarwal et al., 2019^139^	The impact of random and field size MLC positional errors on brain and H&N IMRT plans was assessed.	Random MLC errors up to 2 mm were insignificant. MLC field size errors were significant with up to 10.7% and 15.5% errors observed in EUD for a 2mm error.

**TABLE 5:** Summary of the literature pertaining to the clinical impact of dynamic treatment delivery errors for the VMAT treatment technique.

Publication	Contribution	Key findings/Conclusions
Oliver et al., 2010^145^	Assessed the impact of random and systematic MLC errors on VMAT H&N plans.	There is a linear relationship between PTV gEUD and MLC error for all error types. MLC aperture size errors are more significant than random or aperture offset errors. To maintain PTV70 to within 2% required MLC aperture size errors to be maintained within 0.6 mm
Oliver et al., 2011^146^	Assessed the impact of gantry angle, MU, and MLC (random, aperture offset, and aperture size) errors on VMAT prostate plans.	Gantry angle errors up to 1° were found to be insignificant as were random MU errors. Systematic MU errors caused systematic change in gEUD. MLC random errors were relatively insignificant up to 2 mm. Systematic MLC aperture size errors were more significant than aperture offset errors.
Heilemann et al., 2013^147^	Assessed the clinical impact of MLC errors on prostate and H&N VMAT plans for errors resulting in increased MLC aperture, decreased MLC aperture, and MLC aperture shift.	Errors as small as 1, 0.5. and 3 mm resulted in unacceptable gamma pass rates for MLC errors resulting in MLC aperture increase, decrease, and shift, respectively.
Betzel et al., 2012^152^	Compared the impact of delivery errors on VMAT compared to IMRT. The impact of dose rate, gantry angle, and MLC leaf positions on H&N and prostate plans was evaluated for both VMAT and IMRT.	Systematic MLC shifts were more significant for IMRT than VMAT. IMRT was much more susceptible to gantry angle errors than VMAT. VMAT was more susceptible to dose rate errors, however these needed to be of magnitude outside machine tolerances to be significant.
Nithiyanantham et al., 2015^148^	Assessed the impact of systematic MLC position errors on brain, H&N, and prostate VMAT plans for errors up to 1 mm.	Change in PTV D95% of 1.0%, 1.6%, and 1.9% for brain, H&N and prostate cases respectively for only a 0.3 mm systematic MLC error. Significant dosimetric effect was observed for many cases even with 0.5 mm MLC positional error.
Pogson et al., 2017^150^	Investigated the impact of MLC aperture offset, MLC aperture size, gantry, and collimator angle error generically on VMAT plans.	Significant errors resulting in a ±5 % change in dose metric was consistently observed for ±5 ° collimator angle, ±5mm MLC aperture offset and as small as 1 mm MLC field size error.
Enomoto et al., 2024^149^	Assessed the impact of MLC errors for prostate, lung, spinal, and brain plans and presented treatment site specific MLC positional tolerances.	The MLC positional tolerance for prostate, lung, brain, and spinal plans was found to be: 0.63, 0.34, 1.02, and 0.28 mm respectively.
Terzidis et al., 2024^151^	The impact of random combined MLC position, jaw position, gantry, and collimator angle errors were assessed for prostate, H&N, lung, and gynaecological plans using 3D dose distribution assessment. The assessment was performed for plans with both high and low complexity for each treatment site.	Errors were more significant for complex plans compared to their simple plan counterpart with dosimetric variation greater in the high dose region for complex plans. Dosimetric variations were predominant at the edges of high dose regions that was not apparent with DVH analysis.

**TABLE 6:** Summary of the literature pertaining to the clinical impact of dynamic treatment delivery errors relevant to the SBRT and SRS treatment techniques.

Publication	Contribution	Key findings/Conclusions
Blake et al., 2017^153^	Assessed the sensitivity of SBRT, step‐and‐shoot IMRT and VMAT for lung patients via observed changes to DVH metrics. Errors applied included gantry and collimator rotation errors up to 5° and MLC errors ranging from 1 to 5 mm applied both in the same direction (aperture offset) and in opposing direction (aperture size change).	2 mm systematic MLC errors were observed to result in V100% changes of up to 12%. MLC errors resulting in a change in the aperture size were more significant than those that offset the aperture. MLC errors were more significant to both target coverage and OAR constraints than collimator or gantry errors. Gantry and collimator errors did not significantly impact PTV coverage, but gantry errors could significantly increase spinal cord max dose for step‐and shoot IMRT. VMAT techniques had less patient‐to‐patient variation than step‐and‐shoot IMRT.
Feng et al., 2020^154^	Assessed the sensitivity of SBRT lung plans to MLC errors and investigated the influence of the MU weighting per segment on results.	MLC errors resulting in a change in the aperture size were more significant than those that offset the aperture. The impact of MLC errors was amplified for high‐MU weighting for the affected apertures.
Deng et al., 2022^155^	Assessed the sensitivity of SBRT lung plans to MLC errors using both IMRT and VMAT techniques. Provided MLC tolerances to maintain sufficient plan delivery accuracy.	Random MLC errors were relatively insignificant. For the VMAT technique the MLC offset error, aperture closing error and aperture opening error needed to be restricted below 0.95, 0.32, and 0.38 mm respectively to maintain gEUD of the PTV to within 2%.
Lehmann et al., 2022^6^	Assessed the sensitivity of SBRT spine plans to collimator angle errors, MLC errors, and combined errors.	MLC errors resulting in apertures smaller by 1 mm could result in significant PTV under dosing. MLC errors resulting in apertures larger by 1 mm could result in a significantly hotter hot spot in the spinal cord. Combined errors can be significant to either PTV coverage or spinal cord max dose.
Prentou et al., 2022^157^	Assessed the impact of systematic MLC positioning errors on single isocenter, multi‐target VMAT‐SRS plans.	The impact of MLC errors was strongly correlated with target volume. Dose violations were recorded for two cases for leaf offsets as small as 0.19 mm.
Edward et al., 2025^156^	Assessed the impact of random and systematic MLC errors up to 2 mm magnitude on SBRT spinal plans.	Random errors only resulted in a small impact on PTV dose (0.7%) Whole bank systematic MLC errors resulted in 7.1% PTV dose error on average.

##### 3.3.1.1 IMRT

The studies related to the clinical impact of delivery errors for IMRT are summarized in Table 4. Many of these studies have focused on the impact of MLC errors, which have been shown to be clinically significant.^1^, ^22^, ^131^, ^132^, ^133^, ^134^, ^135^, ^136^, ^137^, ^138^, ^139^ Results vary between studies, likely due to differences in treatment site and planning technique, but systematic errors were consistently reported as having more significant impact than random errors. Errors in the dose‐rate control^133^, ^140^, ^141^, ^142^ and errors in gantry and collimator angle^138^, ^143^, ^144^ have also been investigated with errors at realistic magnitudes found to be generally dosimetrically negligible.

##### 3.3.1.2 VMAT

Many studies now have specifically investigated the impact of delivery errors on VMAT plans. These publications are summarized in Table 5. The impact of MLC errors has been evaluated multiple studies.^145^, ^146^, ^147^, ^148^, ^149^ Results are inconsistent with differences in treatment sites observed^149^ and dependency on TPS and planning techniques also likely. However, MLC errors within standard QA tolerances have been reported to be clinically significant^145^, ^149^ and aperture size errors have been reported to have greater clinical impact than random errors or systematic MLC aperture shift type errors.^145^ The clinical impact to VMAT plans of non‐MLC errors including MUs, gantry angle, and collimator angle has also been investigated with it reported that errors of realistic magnitude were clinically insignificant.^146^, ^150^ One study investigated the impact of combined delivery errors to VMAT plans for multiple different treatment sites.^151^ Besides assessing the impact of the errors on the dose distribution the authors also assessed whether the complexity of the plan for each site affected the impact of the delivery error. The authors observed prominent dosimetric uncertainty at the edge of the high dose region consistent for each treatment site and that this was more profound for plans of higher complexity. The study of Betzel et al.^152^ addressed the question of whether sliding window IMRT or VMAT was more susceptible to delivery uncertainties. It was found that IMRT was significantly more sensitive to gantry angle uncertainties and VMAT was only sensitive to dose rate variations that were larger than typical linac tolerances. The authors concluded that IMRT was more susceptible to delivery uncertainties than VMAT.

##### 3.3.1.3 SBRT/SRS

The studies on the clinical impact of delivery errors for SBRT and SRS techniques are summarized in Table 6. A few of these authors have studied the impact of MLC errors on lung SBRT plans.^153^, ^154^, ^155^ It was consistently observed that MLC errors that resulted in MLC aperture size change were most significant and it was observed in one study that the aperture size needed to be maintained to tighter than standard linac QA tolerances.^155^ It was also observed that the impact of MLC positional error depended not just on the magnitude in the MLC positional offset in each delivery segment but also on the MU‐weighting for that segment.^154^ SBRT spinal treatment has also been investigated for sensitivity to delivery errors^6^, ^156^ with it being observed that collimator error of 1° or all MLC leaves retracted by 0.5 mm could lead to a 10% hotter than expected hotspot in the spinal cord.^6^ In the case of single isocenter Cranial SRS treatments, the impact of MLC positional errors has been investigated.^157^ Change in D95% was found to be linearly dependent on leaf offset at 12%/mm. The impact of offsets was also found to strongly associate with target size with greater impact observed for small targets. In two cases, potentially clinically unacceptable plans were obtained for MLC offsets as low as 0.19 mm.

#### 3.3.2 Linac QA programs

Many proponents of PSQA, particularly log file based PSQA, often caveat that PSQA must be coupled with a robust linac QA program.^126^, ^129^, ^130^, ^135^, ^158^, ^159^, ^160^, ^161^, ^162^ However, there is a question as to whether standard departmental linac programs are comprehensive enough to cover all failures covered by a PSQA measurement. Most linac QA programs are based upon best practice recommendations such as TG‐142,^60^ TG‐198,^61^ and IPEM 81 (2^nd^ ed).^63^ Such recommendations usually just revolve around static testing along with basic dynamic mode testing. The weakness of static testing is that during dynamic treatment deliveries, systems are operated dynamically and it becomes questionable as to whether the static testing covers potential additional failure modes associated with the dynamic operation. Temporal synchronization between dynamic systems is a good example, which if in error could be clinically significant, but is unlikely to be detected in static tests. The reports of NCS 22^30^ and NCS 24^31^ include a wider range of dynamic testing than other linac QA best practice guidance. However, there is a still question as to whether the testing recommended is comprehensive and whether NCS recommendation has been widely adopted. The results of Barber et al.^11^ on IMRT practices in Australia and New Zealand indicated a wide range in dynamic linac QA practice.

Several authors have attempted to develop methodology to address the shortfall in dynamic component linac QA [70–72, 78, 79, 163–165). To provide comprehensive dynamic treatment linac QA a program would likely require both highly effective static system testing as well as highly effective dynamic system testing.

##### 3.3.2.1 Linac QA static testing, best practice, and FMEA

The latest best practice guidance from AAPM on linac QA are those of TG‐198^61^ and MPPG 8.b.^62^ Both documents provide comprehensive recommendations for the static testing of linac components relevant to dynamic treatment delivery. Recommendations are made for all relevant systems including:

1. Mechanical: Verification of isocenters and gantry and collimator readout accuracy at cardinal angles.
2. Dosimetric: Output constancy, including with gantry angle and dose rate, flatness and symmetry, and beam energy constancy.
3. Collimation: MLC positional accuracy, MLC transmission.

Studies have been performed utilizing FMEA analysis on recommended linac QA programs to determine which tests are most important.^163^, ^164^, ^165^, ^166^ Similar to O'Daniel et al.^163^ the risk assessment of MPPG 8.a.^164^ suggested that output constancy and laser localization were the highest priority daily tests and for monthly testing. Output constancy followed by beam profile constancy were determined to be the highest priority annual QA tests. With specific relevance to dynamic treatment techniques the study of Bonfantini et al.^165^ suggested that dose modulation and MLC tests were high priority for linacs performing large dynamic treatment loads while linacs performing treatments with multiple isocenters and/or field junctions couch tests and jaw position were a higher priority.

In 2022, Pearson et al.^166^ proposed a novel Hybrid QA program, which was assessed via FMEA. The Hybrid QA program involved vendor provided tests complemented with conventional QA tests and FMEA was applied to the VMAT, SBRT, conformal, and palliative treatment techniques. The highest priority tests were found to be for MLC dynamic positioning for VMAT and SBRT and then beam position for SBRT and the authors concluded that it would be valuable to perform more rigorous testing of dynamic MLC performance.

Of note, when updating AAPM Medical Physics Practice Guidance with respect to Linac QA, MPPG 8.b.^62^ refrained from including or updating the FMEA risk assessment of MPPG 8.a.^163^ based upon the reasoning of AAPM TG‐100^114^ that risk analysis is unique to each local practice environment.

In the AAPM best practice documents regarding linac QA recommendations are sparse for the assessment of the systems under dynamic control. Only a moving window IMRT delivery and a VMAT PSQA delivery per month is recommended by TG‐198.^61^ For MPPG 8.b. Dynamic Delivery control is recommended with the vendor supplied methodology suggested. Additionally, a sliding window/VMAT holistic test is recommended via a point dose measurement in the target region in phantom for an IMRT/VMAT delivery.^62^ Despite the best efforts of best practice guidance groups, evidence exists for a disparity in methodology for linac QA relevant to dynamic treatments.^11^ In an effort to standardize linac QA procedures and improve their efficiency multiple authors have attempted to develop EPID‐based linac QA suites that meet best practice recommendations.^64^, ^65^, ^66^, ^67^, ^167^ Vendors also now supply linac performance test tools such as Varian's Machine‐Performance‐Check (MPC) and Elekta's Machine QA (also known as AQUA) that have become widely used in linac QA programs.^73^ The utilization of these systems, collectively termed manufacturer‐integrated‐quality‐control (MIQC), are likely to provide significant advantages to local departments in terms of standardization, efficiency and accuracy.^168^ Criticisms of MIQC have generally revolved around their lack of independence from the manufacturer. However, MIQC systems are still being developed and multiple authors have presented methods to improve them.^169^, ^170^, ^171^, ^172^, ^173^ With this development pathway and implementation according to the Hybrid QC principle,^166^ whereby MIQC is routinely verified and supplemented with conventional QA such that independence from the manufacturer is addressed can be expected to result in improved linac QA static testing programs that help negate the need for PSQA measurement. The large uptake of MIQC^73^ has resulted in best practice guidance that has recently been published that provide guidance to allow the safe and effective use of these tools in linac QA programs.^168^, ^174^

##### 3.3.2.2 Dynamic control system testing

Measurement based PSQA has played a role in covering the shortfall of inadequate dynamic control system testing in standard linac QA programs. This shortfall has been identified by multiple authors who have sought to provide solutions. The relevant publications are summarized in Table 7.

**TABLE 7:** Summary of the literature pertaining to linac dynamic control system QA.

Publication	Contribution	Key findings/Conclusions
Ling et al., 2008^70^	First attempt at VMAT dynamic control system QA for Varian linacs using film.	Developed three tests to assess: MLC positioning during arc rotation, dose constancy with dose rate and gantry speed modulation, and dose constancy with varying MLC speed. The tests were widely adopted for Varian systems.
Bedford and Warrington, 2009^71^	First attempt at VMAT dynamic control system QA for Elekta linacs.	Tests developed to assess beam symmetry with dose rate and dynamic MLC tests.
Fredh et al., 2010^175^	Translated the tests of Ling et al., 2008 onto EPID	Tests were successfully translated and automated analysis tools were created significantly improving test efficiency
Van Esch et al., 2011^72^	Attempted to provide comprehensive VMAT dynamic system QA including system synchronization assessment.	Developed three control system tests, one specific to control system synchronization that were not widely adopted.
Jorgensen et al., 2011^181^	Developed three VMAT dynamic system tests using integrated EPID imaging	Developed a picket fence style test for MLC positioning, a dose rate versus gantry speed test and a dose rate versus MLC speed test.
Manikandan et al. 2012^177^	Developed three VMAT dynamic system tests for Elekta linacs.	Developed tests for gantry position vs dose delivery synchronisation, gantry speed versus dose delivery synchronization and MLC speed and positioning tests.
Kaurin et al., 2012^176^	Translated the tests of Ling et al., 2008 onto Elekta EPIDs	Investigated beam profile constancy with dose rate and dosimetry accuracy with MLC reversals and at dose rate changes.
Bedford et al., 2015^182^	Developed a test of MLC vs gantry synchronization during arc delivery	The test had demonstrated sensitivity via deliberately introduced errors.
Barnes et al., 2016^180^	Dynamic assessment of dose rate and gantry speed accuracy during arc delivery	Dose rate and gantry speed were accurately controlled during arc delivery and could be assessed using 2D array device and gantry mount coupled with inclinometer.
Barnes and Greer, 2016^179^	Dynamic assessment of beam symmetry constancy during arc delivery using 2D array detector	Beam symmetry was maintained during arc delivery and that it could be assessed using 2D array device in gantry mount.
Zwan et al., 2016^78^	Dynamic assessment of MLC positioning accuracy during VMAT delivery using cine‐EPID imaging	That MLC leaves were accurately positioned during VMAT delivery and that this could be assessed using the EPID in cine mode.
Zwan et al., 2017^79^	Extended on Zwan et al., 2016 to also dynamically assess beam stability and dose rate control during VMAT delivery using cine‐EPID imaging.	Tests provided for both linac control systems, which are fully stress tested in the assessment. Sensitivity was demonstrated via deliberately introduced errors. Weaknesses identified with gantry angle assessment.

Early methods for dynamic control system QA for VMAT was developed by Ling et al.^70^ for Varian and Bedford and Warrington for Elekta.^71^ The Ling tests were subsequently adopted by Varian and have become highly utilized even with the authors cautioning that there methods were an initial attempt at VMAT linac commissioning and QA. Also these methods have lacked demonstrated detectability to failures. The tests were subsequently transferred from film to EPID^175^, ^176^ and onto Elekta linacs.^177^ Later studies utilized commercial 2D array typed detectors to perform time resolved measurements based on the concept that a time‐resolved measurement was required to properly assess a time resolved delivery with good agreement between plan and measured delivery demonstrated.^178^, ^179^, ^180^ Other studies further pursued the use of integrated EPID imaging for dynamic control system QA^177^, ^181^ extending the scope of testing and updating methodology from initial EPID based methods. A few studies concentrated on the synchronization of dynamic systems as being the key aspect of dynamic control system testing.^72^, ^177^, ^182^ Results suggested that the methods could successfully assure system synchronization.

Low spatial resolution and inconvenience of 2D arrays, impracticalities of synchronization test methods, and absence of temporal resolution of integrated EPID imaging for measuring dynamic systems led to cine‐EPID imaging, whereby measurement can be performed on each EPID frame, to be utilized for dynamic control system QA.^79^ Using cine‐EPID imaging the studies of Zwan et al.^78^, ^79^ presented dynamic control system testing that tested both MLC and dose rate dynamic systems in isolation from one another and with the linac stress‐tested at the extremes of each systems allowed range. Measurements were compared primarily against treatment plans, but also against log files for an additional layer of analysis. Sensitivity of the methods were demonstrated and clinical significance of MLC results, assessed via deviation to the DVH as represented by the measured data, was demonstrated.^78^ By assessing performance over the full range of allowed operation along with some points in between the authors suggested that any clinical plan operating within the same range could be considered assured in terms of the linac delivery performance, hence negating the need for PSQA to assure the linac dynamic control system performance for each individual plan.^79^ Weaknesses of the method to do with the dynamic gantry control system were resolved in a latter publication,^183^ with it suggested that if the methods were performed in tandem then all three dynamic control systems would each be comprehensively assessed. The methods would appear to meet requirements for more rigorous dynamic MLC QA identified via linac QA FMEA analysis^166^ and if combined with an EPID based PSQA type constancy test,^184^, ^185^ to assure that errors from individual systems are not additive to constitute a significant error, then the test regimen could be considered to be comprehensive.

#### 3.3.3 Summary and critical review of the literature

The body of literature on the impact of treatment delivery errors indicate that such errors can be clinically significant. The literature pertaining to FMEA analysis of linac QA recommended tests indicate that such errors do occur, but that their detectability is quite high. Combined, these findings reaffirm the need for comprehensive linac QA. With the advances to static linac QA testing along with the addition of effective dynamic control system test methodologies now available from the literature, it appears that measurement based PSQA may no longer be required for assuring the linac performance for dynamic treatment techniques.

## 4 DISCUSSION

### 4.1 General discussion

Dynamic treatment techniques are more complicated than traditional static beam techniques, however, in terms of deliverability there are still only three sub‐systems that require quality assurance. Therefore, conceptually there is no reason why a return to the original QA paradigm is not feasible. The current literature pertaining to each of the three sub‐systems demonstrates significant development in QA techniques for each since dynamic treatments were first implemented. For each sub‐system, the literature suggests that QA development could be considered to be at a level suggesting that measurement based PSQA is no longer necessarily required. The calculus about the ongoing utility of PSQA should also include the context of the published weaknesses prevalent in many current PSQA processes. The point is not whether sub‐system QA is perfect, but if contemporary sub‐system QA is implemented comprehensively whether PSQA adds any value in failure mode detection.

The important question is not whether measurement based PSQA is required or not. What is important is whether QA programs, in their entirety, detect all of the potentially clinically significant failures. If PSQA is demonstrated to detect such failures, which will not be detected otherwise then PSQA has an important role still to play. However, if this is not demonstrated then there is no need to retain it. As such, QA programs need to be developed first utilizing FMEA type analysis to identify the potentially significant failures. The program should be designed to detect all of the failures and then its ability to do so should be assessed and remediated if required. Such an approach may or may not indicate the need for measurement based PSQA and in all likelihood different departments will reach different conclusions meaning that PSQA could become inconsistently utilized. Such an FMEA study for PSQA is one of the charges of AAPM MPPG20. As such, this study is likely complementary to the efforts of that group.

### 4.2 Weaknesses of contemporary system QA methods

Although the literature demonstrates significant development in QA techniques, arguably to levels making measurement based PSQA redundant, there are challenges to be overcome. Setting appropriate tolerances is a challenge in general for many QA tests, but also for dynamic control system QA in which a failure at just one part of the delivery can be difficult to interpret as to whether it is clinically significant. However, setting meaningful tolerances for PSQA and assuring sensitivity to clinically significant errors has also been challenging and not always demonstrated successfully.^186^ Although QA testing should always strive to detect clinically significant error, PSQA at times has set a low bar in this regard for an alternative system to be considered an improvement. For both PSQA and any alternate program, modern methods are now being more commonly applied in radiotherapy such as FMEA, ROC analysis, and SPC. These methods provide means of improved identification of errors that need to be detected and at what magnitude is significant and can also be used for assessing the effectiveness of QA measures. For both PSQA and time resolved dynamic control system QA the concept of replacing gamma analysis with DVH analysis could potentially provide better clarity as to whether a treatment delivery has clinically acceptable accuracy.

In terms of weaknesses of 3D TPS verification systems, if the models utilize the same input data as the TPS then errors in this data will not be detectable and it is similar for shared algorithm weaknesses. TG‐219^83^ also discusses other limitations including those unknown due to the simplicity of evaluation methods in the literature, handling of patient heterogeneities, and potentially loose tolerances when heterogeneities are not accounted for.

Although log files appear to have significant potential as a data transfer QA tool, a delivery must be performed to generate them. If a user is delivering the plan pre‐treatment to acquire the log file data then with modern EPID‐based PSQA methods^184^, ^185^, ^187^, ^188^, ^189^, ^190^, ^191^ it may not take significant extra time to perform the PSQA measurement as part of the same process providing an additional layer of assessment at little cost. This would likely hold true for hypo‐fractionated treatment plans, where errors in day one deliveries are difficult to recover from. However, the risk calculus may potentially be different for traditionally fractionated plans such that log files could be acquired and analyzed at the first fraction. The concept is also relevant for plan‐of‐the‐day adaptive radiotherapy (ART) whereby a pre‐treatment delivery is not possible.

EPID‐based dynamic control system QA also has limitations beyond the difficulties of tolerances setting. Utilization of the EPID for this purpose is preferable due to its high spatial resolution, strength as a standardized detector and convenience. However, while solutions exist,^183^, ^192^ EPID is not ideal for dynamic gantry angle control QA and cine‐EPID acquisition modes potentially need further development to allow widespread use.

### 4.3 Role of PSQA type measurements ongoing

If, as contended, contemporary QA methods have been developed to the point whereby measurement‐based QA is no longer necessarily required per patient then a relevant question is whether there is still a role for PAQA methods at all? Some contemporary PSQA systems have become highly automated, streamlined, and efficient^184^, ^185^, ^187^, ^188^, ^189^, ^190^, ^191^ such that they aren't the same resource burden as other or previous techniques. As such, these techniques could provide an extra layer of testing at minimal cost to provide QA redundancy supplementing a comprehensive system QA program. It is plausible that such methods be developed to the point whereby they are both highly efficient and provide a comprehensive overall process check of patient plan deliverability. If combined with modern sub‐system QA techniques then the role of the PSQA measurement could be to act as an overall process check of each specific patient plan's deliverability providing assurance of clinical acceptability, while the role of sub‐system QA is to identify the cause of fails if they do occur and potentially pre‐empt fails via the predictive QA concept^171^, ^193^, ^194^ whereby analytics are applied to longitudinal QA data to forecast when a parameter will reach a clinically unacceptable state.

Another potential pathway is for PSQA methods to be retained but not performed on every patient plan. This would align with the approach of NCS, 22^30^ and NCS 24.^31^

### 4.4 Relevance for further advances in treatment technique

If measurement based PSQA were to be withdrawn from per patient use then the methods would likely retain a role in new technique and technology commissioning and in dosimetric auditing, credentialing, and benchmarking. It could well prove advisable to run PSQA for a defined set of initial patients until the treatment planning process has been bedded in and there is confidence that it is optimal. PSQA would also likely be retained for a period to be run in parallel with any non‐PSQA program until sufficient confidence had been obtained as to it not being required. In terms of new techniques, while some such as online‐Adaptive RT, measurement based PSQA is not favorable, while others there is still a potential role. The uptake of motion management techniques provides new treatment data that needs to be assured and also requires changed operations of the treatment machines that need to be considered. The linacs ability to deliver accurately in the presence of beam holds has, in a different context, historically been questioned.^133^

### 4.5 Recommendations

From the findings of this study it is the belief of the author that routine sub‐system QA techniques have developed to the level that measurement‐based PSQA is not necessarily required for assuring treatment deliverability. However, this is likely local practice dependent and at local responsibility and requires careful consideration to be adopted. The key question is whether PSQA adds value in failure mode detection. Although this review was not commissioned to provide guidance, a cautious process for discontinuation of measurement based PSQA could be:

1. Comprehensive TPS commissioning compliant with AAPM MPPG 5.b.^80^
2. Implementation of a sophisticated TPS dosimetry check system with full compliance with AAPM TG‐219.^83^
3. A comprehensive data transfer QA program in full compliance with AAPM TG‐201^108^ complemented with a comprehensive chart rounds program fully compliant with AAPM TG‐275^125^ and fraction 0 (hypofractionated) or fraction 1 (standard fractionation) log file analysis.
4. A comprehensive linac QA program fully compliant with AAPM TG‐198^61^ supplemented with demonstrated effective linac dynamic control system QA.
5. FMEA analysis to identify the local clinically relevant deliverability failure modes. The FMEA analysis of O'Daniel et al.^9^ could be used to guide this process, but as per AAPM TG‐100^114^ such risk analysis should be performed locally, potentially with guidance from vendors as suggested in AAPM TG‐332.^174^
6. Demonstrated detectability of each significant failure mode by the QA program at an appropriate frequency. In the event of clinically significant failure modes being identified which are not demonstrated to be detected by the QA program then PSQA techniques should be utilized that can detect these failures.

An important distinction is that log files are recommended for verifying control point data integrity (i.e. confirm that the linac received the correct treatment plan data), but they are recommended against for linac dynamic control system QA (i.e. confirming that the linac physically delivered the plan accurately). This is due to lack of independence^73^ of log file data from the systems under investigation and demonstrated insensitivity of log files to clinically significant failure modes.^74^, ^75^, ^76^, ^77^

## 5 CONCLUSIONS

Measurement based PSQA has become a significant burden on radiotherapy departments and evidence exists for its ineffectiveness at detecting the clinically relevant delivery errors which is its purpose. The literature suggests that the advances in QA techniques for each of the three sub‐systems of deliverability may allow for the safe removal of measurement based PSQA from standard practice. However, this is likely local practice dependent and requires a comprehensive sub‐system QA program with demonstrated detectability to all clinically significant failures. The role of PSQA ongoing should be for the safe introduction of new techniques and/or, as it was always intended, to fill gaps in sub‐system QA program error detection capability. The decision and responsibility to discontinue PSQA should be taken locally in the context of local practice and at local responsibility and should only be done with careful consideration of the risks.

## AUTHOR CONTRIBUTIONS

Michael Barnes was sole investigator on the study and therefore had the original idea for the study, scoped the project, performed the literature review, and wrote the manuscript.

## ETHICS STATEMENT

No human nor animal studies were performed as part of this study.

## FUNDING INFORMATION

The author received no funding for this study.

## CONFLICTS OF INTEREST STATEMENT

The author declares tno conflict of interest.

## Data Availability

Not applicable.
